# Case report: Clinical and neuroradiological longitudinal follow-up in Leukoencephalopathy with Calcifications and Cysts during treatment with bevacizumab

**DOI:** 10.3389/fneur.2023.1245014

**Published:** 2023-09-20

**Authors:** Elena Scaffei, Bianca Buchignani, Rosa Pasquariello, Paola Cristofani, Raffaello Canapicchi, Laura Biagi, Flavio Giordano, Emanuela De Marco, Yanick J. Crow, Roberta Battini

**Affiliations:** ^1^Department of Neuroscience, IRCCS Stella Maris Foundation, Pisa, Italy; ^2^Department of Neuroscience, Psychology, Drug Research and Child Health NEUROFARBA, University of Florence, Florence, Italy; ^3^Department of Neurosurgery, Meyer Children's Hospital IRCCS, Florence, Italy; ^4^Paediatric Oncology and Haematology Department, Santa Chiara Hospital, Azienda Ospedaliero Universitaria Pisana, Pisa, Italy; ^5^MRC Human Genetics Unit, Institute of Genetics and Cancer, University of Edinburgh, Edinburgh, United Kingdom; ^6^Laboratory of Neurogenetics and Neuroinflammation, Institut Imagine, Paris Descartes University, Paris, France; ^7^Department of Clinical and Experimental Medicine, University of Pisa, Pisa, Italy

**Keywords:** LCC, leukoencephalopathy, cysts, calcifications, Labrune syndrome, MRI, bevacizumab, inherited white matter disease

## Abstract

Leukoencephalopathy with Calcifications and Cysts (LCC) is a rare genetic microangiopathy exclusively affecting the central nervous system caused by biallelic mutations in *SNORD118*. Brain magnetic resonance imaging (MRI) is often diagnostic due to the highly characteristic triad of leukoencephalopathy, intracranial calcifications, and brain cysts. Age at onset, presentation and disease evolution can all vary, ranging from pauci-symptomatic disease to rapid evolution of signs with loss of motor and cognitive abilities. No specific therapies for LCC are currently licensed. According to the literature, bevacizumab might represent an effective modality to improve the clinical and MRI features of the disease. However, uncertainty remains as to the true efficacy of this approach, when to begin therapy, appropriate dosing, and the consequences of drug withdrawal. According to CARE guidelines, we describe the long-term clinical and neuro-radiological follow-up of a 10-year-old child with LCC. We report disease evolution following repeated cycles of treatment with bevacizumab. Our case report suggests that repeated cycles of bevacizumab might effectively modify disease progression, possibly indicating a time-dependent effect.

## Introduction

Leukoencephalopathy with calcifications and cysts (LCC), also referred to as Labrune syndrome, is a rare disease, which was described for the first time in 1996 (1) and has only recently been genetically characterized (2, 3). LCC is an inherited white matter disease caused by biallelic mutations in *SNORD118* (OMIM:616663), encoding the box C/D small nucleolar ribonucleic acid (snoRNA) U8 (3), a non-coding RNA vital for ribosomal RNA homeostasis. While the precise role of SNORD118 in ribosome biogenesis and maturation remains uncertain, the impact of its deficit on brain function is clear (4). From a pathological point of view, LCC is an exclusively brain microangiopathy, where cerebral small blood vessels exhibit angiomatous-like features and Rosenthal fiber deposition with perivascular mineralization and minimal inflammation (5). Macroscopically, neuroimaging studies typically reveal the pathognomonic triad of leukoencephalopathy, calcifications, and cysts. LCC shares a remarkable overlap with the neuroradiological phenotype of Coats plus syndrome, a systemic diffuse microangiopathy affecting not only the brain but also the retina, gastrointestinal tract, and bone, caused by biallelic mutations in *CTC1*. The molecular pathology and clinical spectrum of 64 LCC patients were recently described, underlining the marked heterogeneity in disease presentation and progression, only partially explained by genotype–phenotype correlations (6). Indeed, in this most recent series, age at presentation varied from 3 weeks to 67 years, and clinical features can range from purely neurological signs to psychiatric manifestations (4, 7).

Although rare, as a slowly progressive chronic illness, LCC represents a major health burden to affected individuals and their families. No standard treatment paradigm for LCC has yet been described, with currently available options aimed at the symptomatic management of disease complications such as motor impairment, epilepsy, and intracranial hypertension. Thus, as one example, in patients with progressive symptoms and/or growing lesions on imaging studies, cystic surgical procedures (cystic puncture, cystic resection, cysto-ventriculoperitoneal shunting) are used to manage intracranial hypertension (8).

Interestingly, starting from the previous application in unilateral Coats disease (9), three reports have described the use in LCC of bevacizumab, a monoclonal antibody that binds to VEGF with high specificity, thereby blocking VEGF-mediated signaling pathways and thus angiogenesis (10–12). Albeit single case reports, a possibly encouraging effect of treatment, both in relation to clinical progression and neuroradiological appearance, has been suggested using this approach. However, due to the rarity of the disease, a knowledge gap exists in relation to the efficacy of this approach. Here, we describe an apparently convincing response to bevacizumab in a patient with typical LCC, on both clinical and neuroradiological features.

### Case description

**Figure 1:**
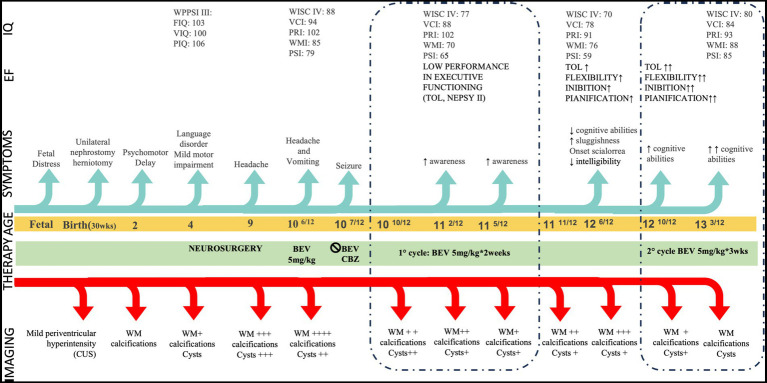
Clinical timeline. CUS: Cranial Ultrasonography, EF: Executive Functioning, FIQ: Full IQ, IQ: Intelligence Quotient, PIQ: Performance IQ, PRI: Perceptual Reasoning Index, PSI Processing Speed Index TOL: Tower of London, VCI: Verbal Comprehension Index, VIQ: Verbal Index, WM: White Matter, WMI Working Memory Index. Age is given in years. Modifications in neuroradiological features are shown as ‘+’ in consideration of the involvement of WM, calcifications, and cysts: + mild ++ moderate +++ severe ++++ extremely severe. WM assessment refers to represent severity of leukodystrophy and not its topology. Similarly, cysts evaluation refers to qualitative assessment.

A three-year-old Italian boy was referred to IRCCS Fondazione Stella Maris because of psychomotor delay (timeline of major clinical and neuroradiological assessments in Figure 1). Family history was not contributory. Prenatal history revealed fetal distress due to unilateral hydronephrosis and abruptio placentae, resulting in premature birth at 30 weeks’ gestation and NICU recovery. Unilateral nephrostomy and herniotomy were performed in the first weeks of life, and mild periventricular hyperechoic lesion was detected with cranial ultrasonography. Due to the prematurity, an active surveillance of neurodevelopmental outcome was started, allowing for the charting of a major delay in postural and communicative milestones. At the age of 2 years brain magnetic resonance imaging (MRI) was performed, revealing a non-specific pattern of leukoencephalopathy and brain calcifications. On initial neurological examination, the child presented mild, asymmetrical pyramidal signs. Metabolic analyses (specifically organic acids, serum and urinary amino acids, lactate levels and acylcarnitines), and TORCH antibodies titles were normal, as was genetic testing for mutations in *COL4A1*. Short-term clinical follow-up highlighted the presence of a language disorder, mild motor impairment and normal intellectual functioning (Wechsler Preschool and Primary Scale of Intelligence: Full Scale IQ 103, Verbal IQ 100, Performance IQ 106). Cranial MRI was repeated at the age of 4 years, highlighting a progression of neuroradiological features, and now demonstrating the appearance of small parietal cysts. Thus, having excluded metabolic and infectious etiologies, an underlying genetic early-onset leukodystrophy was suspected, with the pattern of white matter alterations (mainly localized posteriorly and asymmetrically) helping in the differential diagnosis. Of note, other inherited causes tend to demonstrate a more prominent temporal involvement (mitochondrial leukoencephalopathies), frontal involvement (Alexander disease) or both frontal and temporal involvement (Aicardi-Goutières syndrome). Furthermore, the presence of calcifications helped to exclude a diagnosis of Vanishing White Matter Disease and Megalencephalic leukoencephalopathy with subcortical cysts. Notably, the possibility of LCC became more compelling as cystic elements evolved with neuroradiological follow up, thereby highlighting the characteristic triad of leukoencephalopathy with cyst and calcifications (13). Testing of *SNORD118* identified compound heterozygosity for an n.103G > A apparently *de novo* variant, and a maternally inherited n.39G > C variant both of which have been previously reported as mutations (14).

**Figure 2:**
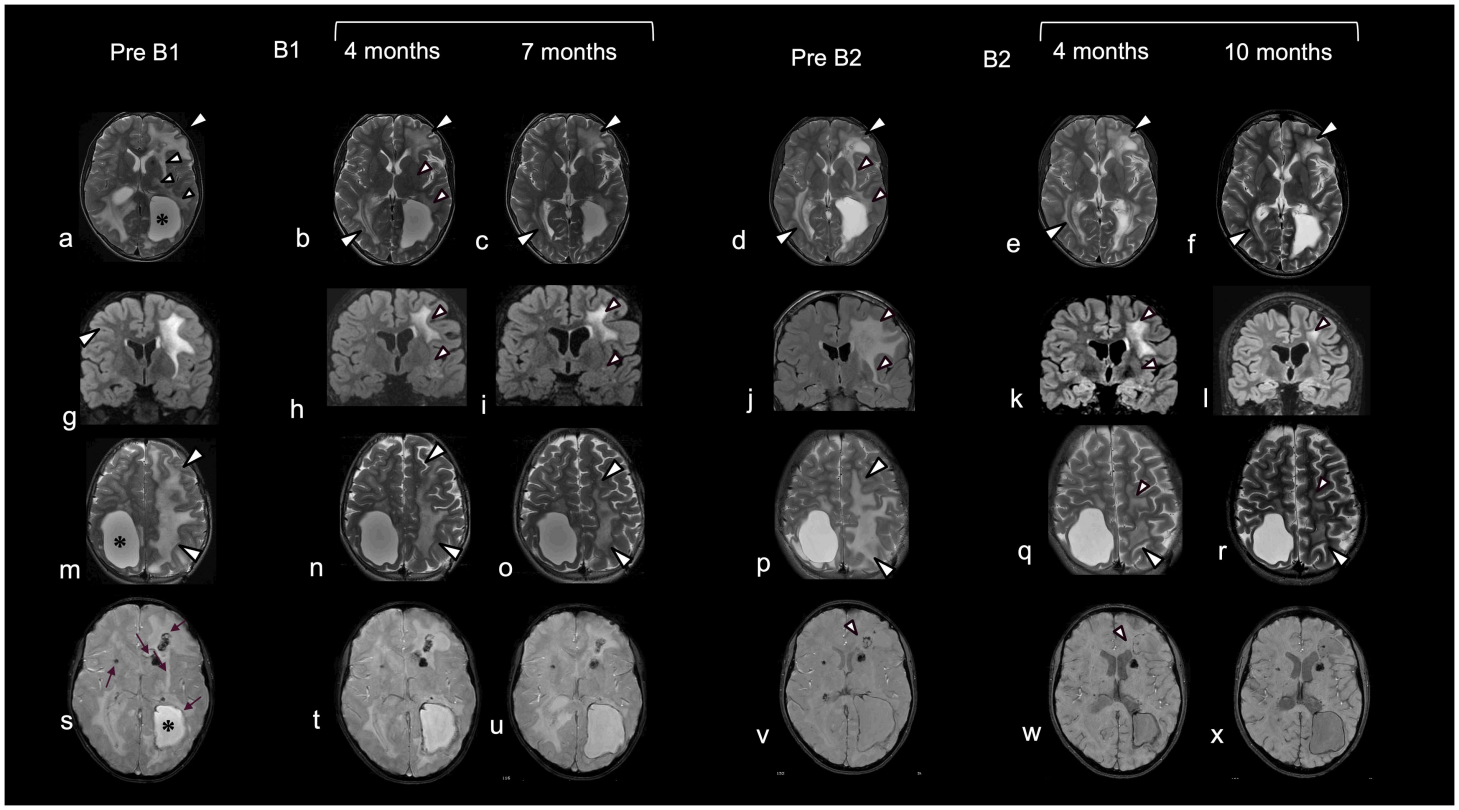
Neuroradiological data pre- and post-treatment with bevacizumab. Comparative brain MRI before and after the first and second cycle of treatment with bevacizumab. Before the initiation of treatment (pre B1) T2-weighted images (a,g,m) showed extensive and asymmetric hyperintensity of the periventricular and deep white matter, with involvement also of the juxtacortical U-fibers (a,m: white arrowheads) more extensive on the left, with lobar macrocysts (*) delimited by hypointensity in T2 Star Weighted ANgiography (SWAN) (s) due to calcium and hemosiderin deposition. Additional microcysts, microcalcifications and microdeposits of hemosiderin were present in the periventricular white matter, around the walls of the small cysts, and in the region of the putamen on the right and the caudate on the left (s: arrow). Four (b,h,n) and 7 months (c,i,o) after starting a first cycle of treatment there was a progressive reduction in the extent of the signal alteration, which first affected the white matter of the U fibers (b,c,n,o: white arrowheads), external capsular claustrum and posterior limb of the internal capsule and periventricular white matter (small white arrowheads). MRI images after discontinuation of treatment (pre B2) showed a reappearance of signal alterations in the same regions in which regression was observed (d,j,p vs a,g,m: white arrowheads). After initiation of the second treatment cycle, progressive improvement of signal alterations was observed in the same regions as after the first cycle (e,f,k,l). In addition, a reduction of signal hypointensities in SWAN sequences was observed in the frontal white matter on the left, around, and in proximity to, the cyst wall (v,w,x: arrowheads). Note the apparent decrease in abnormal white matter volume after treatment (c,o; f,r).

The child’s clinical and neuroradiological status remained substantially stable until the age of 9 years, when he began to complain of recurring headaches. Further MRI revealed an enlargement of brain cysts’ volume, resulting in compression of the lateral ventricles, and a worsening of the extent of white matter signal changes particularly involving the deep and juxtacortical regions. As a result, he underwent neurosurgical stereotactic drainage of three cysts in the same procedure performed with Leksell-G Frame® (Elekta, Stockolm, Sweden) coupled with Neuromate Neuroinspire® Stereotactic Robot (Renishaw-Mayfield SA, Nyon, Switzerland). In the first weeks after surgery, the child recovered from headaches, and a further cognitive evaluation showed an improvement over an assessment performed 2 years earlier. However, 1 month later the child re-presented with clinical symptoms suggestive of intracranial hypertension. Brain imaging again revealed an increase in cysts size and a worsening of white matter lesions (Figure 2 pre B1). Further, T2-weighted images showed extensive and asymmetric hyperintensity of the periventricular and deep white matter, with involvement also of the juxtacortical U-fibers, and the presence of lobar macrocysts, microcysts and microcalcifications in the periventricular white matter.

Given the relapse of neuroradiological features despite recent surgical intervention, and the encouraging results described in a single case report (10), bevacizumab was started at age 10 years, administered at a dose of 5 mg/kg intravenously every 2 weeks. After three injections, the child presented with symptomatic focal seizures, which were easily controlled by carbamazepine. Bevacizumab was discontinued, and then restarted after 2 months (first cycle of bevacizumab: B1), without any recurrence of seizures or other side effects. An improvement of MRI features was recorded after 1 month of treatment, with a reduction of white matter signal alteration particularly in the left hemisphere.

**Figure 3:**
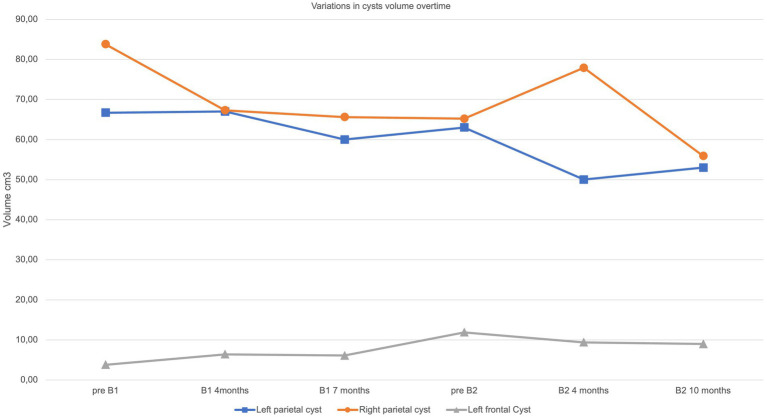
Volumetric variation of macrocysts. Graphical representation overtime of cysts’ volume according to timepoints described in main text and in Figure 2.

By the fourth month of treatment, a further improvement in the neuroradiological appearance was observed, with a reduction in white matter involvement in the juxtacortical regions, in the external capsular claustrum and in the posterior limb of the internal capsule and periventricular regions (Figure 2 B1). Modifications in cysts’ volume and shape were also recorded (Figure 3). These neuroradiological changes were accompanied by an improvement in awareness. After 7 months of therapy, further neuroradiological and clinical improvements were documented, albeit to a lesser degree than during the first months of treatment (Figure 2 B1). According to the report by Fay et al. (10), bevacizumab was stopped after 12 months. In our case, MRI follow up at one and 8 months after treatment cessation showed a worsening in neuroimaging (Figure 2 pre B2). Interestingly, the areas of change involved the same brain regions which had previously improved. This neuroradiological deterioration was accompanied by a loss of cognitive abilities, increased sluggishness, sialorrhea and reduced intelligibility of speech. For these reasons, a second cycle of bevacizumab (B2) was started. In order to reduce the number of hospitalizations, the drug was administered every 3 weeks with good adherence and tolerability. After 4 months of treatment, MRI revealed an improvement in the appearance of the white matter, with the same topological distribution observed previously (Figure 2 B2).

Further marked modifications were observed after 10 months of treatment, including a reduction of white matter abnormalities, a mild enlargement of the periencephalic subarachnoid space and variation in cysts’ volume (Figures 2 B2, 3). No changes were observed in contrast enhancement, and no new calcium deposits were noted. Signal inhomogeneity in the left frontal lobe, previously identified as an area of atypical vascularization with hemosiderin deposition, was also reduced. Similarly, a new and more significant clinical improvement was noted, with reduction of sialorrhea and increase in awareness; an extensive neuropsychological assessment performed at this time indicated an improvement in executive functioning and processing speed.

## Discussion

To the best of our knowledge, we report the fourth case of LCC treated with bevacizumab, but notably the first to receive repeated cycles of pharmacological treatment. We consider the following as important points of discussion. Firstly, previous reports described a single 12-month period of treatment (10–12), associated with apparent clinical improvement in two patients (10, 12), even if only moderate in one (12). Of note, clinical improvement in these two cases appeared to plateau after 6 months of therapy. Conversely, our patient showed an ongoing clinical improvement, especially in neuropsychological abilities, more prominent during the second cycle of therapy, despite a reduced frequency of administration of bevacizumab compared to the first cycle. These observations lead us to suggest that further studies are warranted to determine if there could be a cumulative effect of therapy, or a therapeutic window-dependent effect. We also speculate that genotype could modulate pharmacological response, noting that the patient homozygous for a n.8G > C mutation in *SNORD118*, which is rare and can be associated with a more severe clinical phenotype (3), showed no clinical response to treatment (11).

Secondly, evidence of a neuroradiological response to bevacizumab is currently difficult to determine from the cases published to date, with an absence of regression of brain lesions in the single-reported adult patient (12) compared to the two younger subjects (10, 11). Indeed, the age of the treated patients in these reports ranged from 19 months to 53 years, and it can be speculated upon that the initiation of therapy at a younger age might be associated with a better clinical outcome. Consistent with previous reports, we observed a regression in white matter disease but only a slight modification in cyst volume, with calcifications remaining substantially unchanged over time. Possibly of note, we recorded a reabsorption of hemosiderin depositions.

Finally, we observed a recurrent pattern in the topology of bevacizumab responsiveness. Thus, during both cycles of therapy, white matter changes began in the same regions, possibly suggesting a differential sensitivity of LCC brain lesions to VEGF-inhibition. Since LCC is a progressive and multiphasic disease, several stages may be specifically sensitive to pharmacological intervention, differentiating brain areas in a latent or active stage of the disease. Future neuropathological studies should be addressed to understand if there is recurrent pattern in VEGF-response. If so, the therapeutic response may not only be influenced by the age of the patients, but also by the number of cells in a “latent” stage, which enables them to respond to treatment.

Limitations and challenges should be addressed. Firstly, the timeline of neuroradiological follow-up was not strictly homogeneous considering the relative invasiveness of MRI exams. Furthermore, in distinction to leukodystrophies such as Metachromatic Leukodystrophy (15), a more rigorous and standardized scoring system of MRI images is not currently available for LCC in order to properly assess disease severity. Thus, our neuroradiological evaluation is qualitative, with the timeline was based on clinical evolution. To facilitate an interpretation of the MRI pattern as reliable as possible, all assessments were performed by the same neuroradiologist. Of note, an adjustment to the therapeutic protocol that we employed was made during follow-up, in order to meet the needs of the patient and thereby increase compliance. This regimen should be taken into account in future inter-patient assessments. Clearly, further studies are needed to optimize therapeutic protocol.

In conclusion, our report adds significant knowledge about the therapeutic effect of bevacizumab in LCC. Even though a full recovery was not observed, we consider likely that bevacizumab treatment slowed disease progression in our case. We interpret these data to support the further investigation of the efficacy of bevacizumab in the treatment of this devastating disorder, ideally in the context of a controlled clinical trial. Such experimental studies should include a consideration of when to begin therapy, the dosing to be used, and the consequences of drug withdrawal. In terms of future clinical practice and research, we would recommend the collection of data in a centralized registry in order to better determine the natural history of LCC.

### Patient perspective

During all treatment, assessments and follow up, the patient and his parents were involved in supportive dialog both with clinicians and with a clinician-trained psychologist. The child, who was initially afraid of the procedure, gradually became more tolerant both to the injections and to the frequently requested MRIs. To date, he has not complained of any side effects, with the timing of such procedures tailored to minimize disruption to his home and school life.

## Data availability statement

The datasets for this article are not publicly available due to concerns regarding participant/patient anonymity. Requests to access the datasets should be directed to the corresponding author.

## Ethics statement

The studies involving humans were approved by Comitato Etico Pediatrico Regione Toscana Meyer. The studies were conducted in accordance with the local legislation and institutional requirements. Written informed consent for participation in this study was provided by the participants’ legal guardians/next of kin. Written informed consent was obtained from the individual(s) for the publication of any potentially identifiable images or data included in this article.

## Author contributions

ES and BB: writing-original draft preparation. ES, BB, RP, and PC: writing-review and editing. RP, LB, and RC: elaboration of figure. RB, YC, EM, and FG: supervision. RB and LB: funding acquisition. All authors have participated in conceptualizing the case report, read, and agreed to the published version of the manuscript.

## Funding

This work was partially supported by the Italian Ministry of Health (Grant RC 2023) to IRCCS Stella Maris Foundation (ES, BB, PC, RC, LB, RP, and RB). YC acknowledges financial support from the MRC (MRC Human Genetics Unit: MC_UU_00035/11).

## Conflict of interest

The authors declare that the research was conducted in the absence of any commercial or financial relationships that could be construed as a potential conflict of interest.

## Publisher’s note

All claims expressed in this article are solely those of the authors and do not necessarily represent those of their affiliated organizations, or those of the publisher, the editors and the reviewers. Any product that may be evaluated in this article, or claim that may be made by its manufacturer, is not guaranteed or endorsed by the publisher.
